# The Association Between Cardiorespiratory Fitness Directly Assessed by the Cardiopulmonary Stress Test and the Perception of Stress

**DOI:** 10.3390/jcm14197120

**Published:** 2025-10-09

**Authors:** Gianluigi Oggionni, Marcello Rizzi, Giuseppina Bernardelli, Mara Malacarne, Massimo Pagani, Daniela Lucini

**Affiliations:** 1 Exercise Medicine Unit, IRCCS Istituto Auxologico Italiano, 20135 Milan, Italy; gianluigi.oggionni@auxologico.it; 2 BIOMETRA Department, University of Milan, 20129 Milan, Italy; marcello.rizzi@unimi.it (M.R.); mara.malacarne@unimi.it (M.M.); 3 DISCCO Department, University of Milan, 20122 Milan, Italy

**Keywords:** fat mass, lifestyle, prevention, well-being

## Abstract

**Background/Objectives:** Cardiorespiratory fitness (CRF) represents a strong and consistent predictor of mortality among adults. It is ideally expressed as the maximum or peak rate of oxygen consumption per kilogram of body mass (VO_2max_) determined by the cardiopulmonary exercise test (CPX). Variance in CRF is mainly attributable to genetics and physical training; nevertheless, strong behavioral and socioeconomic confounders need to be considered. Among those, psychosocial stress may play an important role. Some papers show an association between low CRF and chronic stress conditions; nevertheless, CRF is generally estimated by indirect assessment and not directly measured by CPX. **Methods**: CRF was directly assessed by performing a CPX in 145 consecutive subjects (56 male, 89 female) (age 19–65 years) who attended our Exercise Medicine unit for health check-ups. Weekly total volume of physical activity (PA) was evaluated using a validated questionnaire (IPAQ); perceptions of stress, fatigue, and somatic symptoms were assessed using a self-administered questionnaire. **Results:** VO_2max_ was negatively correlated with perception of stress (*p* = 0.03), fatigue (*p* < 0.001), and somatic symptoms (*p* < 0.001); as expected, it was positively correlated with the weekly volume of PA (*p* < 0.001). This link was further evidenced by the observation that subjects who did not meet the PA goals as indicated by WHO guidelines presented a higher perception of stress, fatigue, and symptoms, as compared to physically active subjects. **Conclusions:** This direct link might, on the one hand, corroborate the role of exercise as a tool to manage stress and, on the other hand, focus on the role of stress as a possible determinant of CRF.

## 1. Introduction

There is no doubt regarding the role of cardiorespiratory fitness (CRF) in determining health and reducing cardiometabolic risk [1,2,3,4,5,6,7,8]. CRF reveals the interplay between the pulmonary, cardiovascular, and muscular systems in transporting oxygen from the atmosphere to the exercising muscles [9]; it is inversely related to all-cause mortality and the incidence of several diseases, ranging from cardiometabolic diseases [1,2,3,4,5,6,7,8] to cancer [10] and depression [11]. Moreover, its improvement is linked to a better prognosis of several conditions [12,13,14]. The link between CRF and health is so robust that it is nowadays considered a potentially stronger predictor of mortality than established risk factors [8].

CRF is ideally expressed as the maximum or peak rate of oxygen consumption per kilogram of body mass (VO_2max_), which is determined by cardiopulmonary exercise testing (CPX) [9]. However, this test is costly and requires special equipment and expertise. In clinical practice, when it is not feasible or appropriate to perform a CPX test, a simple ECG stress test can approximate exercise capacity, usually expressed in estimated metabolic equivalents of task (METs) [2,4], representing multiples of the basal oxygen consumption rate at rest [7,8,9,15]. Additionally, indirect methods, such as exercise and non-exercise prediction equations [16,17,18] or questionnaires assessing exercise volume [19], are often used in both clinical and research settings.

Variance in CRF is mainly attributable to genetics and physical training [20]; nevertheless, strong behavioral and socioeconomic confounders need to be considered [6]. Among those, psychosocial stress may play a role [21]. The scientific literature is rich in papers showing an association between low CRF (mainly assessed by indirect measures) and conditions characterized by high stress perception [14,22,23], such as burn-out syndrome [24,25], depression and anxiety [23,26] or prolonged military exercise [27]. Moreover, physical inactivity was well demonstrated to be linked to increased stress perception [28,29,30,31,32]. Stress is gaining significant importance in the medical field [33,34,35,36,37], as it negatively affects well-being and health [36,37], representing an independent risk factor for major chronic diseases such as cardiovascular [33,34,35,36,37], metabolic [35], and oncological [34] conditions. Therefore, it is increasingly crucial to develop sustainable clinical strategies capable of preventing or managing stress. In this context, it is particularly interesting that aerobic physical exercise (the most important modifiable determinant of CRF) may foster a reduction in chronic stress perception and enhance well-being [26,38,39,40,41,42]. Many scientific papers and meta-analyses have addressed the differences in types of exercise and their impact on stress management. A significant cross-sectional study [43] showed that many exercise types were associated with a lower mental health burden than not exercising; indeed, the strongest associations were observed for popular team sports, cycling, and aerobic and gym activities, evidencing also that “more exercise was not always better”, underlying the importance of exercise volume (the combination of intensity, duration and frequency). Another large Korean study [44] showed a dose–response relationship between exercise frequency and reduced perceived stress; additionally, a broad Danish study found that physical inactivity was strongly associated with higher perceived stress [45]. Also exercise modalities such as functional training, dance, gym activities [46,47], or multicomponent exercise, for instance tai chi or yoga, were shown to improve quality of life/mood and reduce stress, in particular those which add the modulation of breathing [48] to aerobic/flexibility/strength modality of exercise [46,49,50,51].

Consequently, many guidelines and institutions recommend exercise as a primary tool for stress management and well-being promotion [52,53,54,55], highlighting a bidirectional relationship between stress and physical activity that needs to be acknowledged [22,56].

It is, in fact, important to consider that the perception of a high stress level may represent a barrier to performing exercise or to reaching/maintaining elevated sport performances [51]; thus, stress management techniques are now considered for elite athletes [51].

Nevertheless, to the best of our knowledge, the scientific literature contains few papers that show a correlation between CRF, directly measured by VO_2max_, and stress perception indices. Demonstrating this direct link could, on the one hand, support the idea that exercise is a useful tool for managing stress and, on the other hand, focus on the role of stress as a potential determinant of CRF.

The aim of this study was to study the differences in stress perception in two groups of subjects attending our clinic for health check-ups, characterized by different exercise training levels (assessed by the IPAQ, which establishes weekly exercise volume [57]), considering physically active subjects (who reach the physical activity goals as suggested by the World Health Organization (WHO)’s physical activity guidelines [56]) and inactive subjects (those who do not meet WHO guidelines), hypothesizing that physically inactive subjects would be characterized by high stress perception and low CRF. The secondary aim was to study the association between CRF, directly assessed by CPX, and the perception of stress and somatic symptoms.

## 2. Materials and Methods

### 2.1. Study Design

This observational, cross-sectional study involved 145 consecutive subjects (56 male; 89 female) who attended our Exercise Medicine unit for health check-ups. Inclusion criteria were as follows: age 18–65 years; absence of major diseases (myocardial infarction, arrhythmias, diabetes, and oncological or psychiatric disease); and not being a professional athlete. All participants gave their informed consent. The protocol of this observational study followed the principles of the Declaration of Helsinki and Title 45, US Code of Federal Regulations, Part 46, Protection of Human Subjects, Revised 13 November 2001, effective 13 December 2001. It was approved by the local Institutional Ethics Committee (Ethics Committee Istituto Clinico Humanitas, 13 October 2015 and Ethics Committee Ospedale L.Sacco, Prot 651/09/70/AP, 16.12.09). The subjects acknowledged that they could not be identified via this paper and that the authors had fully anonymized their data.

The population of our study was subdivided into two groups, considering the total weekly physical activity volume of moderate and vigorous exercise: those (n = 87 group ABOVE) reaching the physical activity goals as suggested by the latest WHO guidelines [48] corresponding to 150 min/week of moderate activity, 75 min/week of vigorous activity, or a combination of both (≥600 (MET·min/week), considering the total weekly physical activity volume of structured exercise METs MV), and those (n = 58 group BELOW) who did not reach the physical activity goals (Below 600 MET·min/week).

All subjects underwent the following assessments:

### 2.2. Clinical Assessment

- Medical history, a standard physical examination, and anthropometric data were collected, including weight, height, waist circumference (WC), body mass index (BMI), and resting hemodynamic parameters. BMI was calculated according to the standard formula [58]. Blood pressure and heart rate were measured in a seated position after at least 5 min of rest, using a validated automated oscillometric device (Omron Healthcare, Kyoto, Japan). The mean of the two consecutive measurements was reported.
- Venous blood samples were collected in the morning after an overnight fast (≥8 h) and analyzed for total cholesterol, HDL cholesterol, triglycerides, and fasting glucose, using standard enzymatic colorimetric assays (Roche Diagnostics, Mannheim, Germany).
- BIA (Bioelectrical Impedance Analysis; BodyStat Quadscan 4000, BodystatR Quadscan 4000, Body Stat Ltd., Isle of Man, British Isles, UK) was employed to estimate the percentage of fat mass (FM) and of free fat mass (FFM) using the proprietary equation provided by the manufacturer [59]. Measures were obtained under standardized conditions (fasting state, voided bladder, no exercise in the preceding 12 h), as recommended in the BIA assessment protocols [60].

### 2.3. Aerobic Fitness

Aerobic fitness was assessed by maximal oxygen uptake (VO_2max_), determined during a standardized incremental cardiopulmonary exercise test (CPX) performed on a cycle ergometer (Ergoline GmbH, Bitz, Germany). After a 2 min unloaded warm-up, the workload was gradually increased in a ramp protocol (10–20 W/min, tailored by sex, age, and fitness level) until the participant reached voluntary exhaustion. Participants were instructed to maintain a pedaling cadence of 60–70 rpm throughout the test. Expired gases were collected breath-by-breath using a calibrated metabolic cart (Vmax Encore, Viasys, CareFusion, San Diego, CA, USA). Oxygen uptake (V′O_2_) and carbon dioxide output (V′CO_2_) were calculated using standard algorithms based on the Haldane transformation [61]. VO_2max_ was defined as the highest 30 s average VO_2_ recorded during the test. Maximal effort was confirmed if at least one of the following criteria was met:

- Respiratory exchange ratio (RER) ≥ 1.10;
- Plateau in VO_2_ despite increased workload;
- Attainment of age-predicted maximum heart rate [62].

A continuous 12-lead ECG (Cardioline, Cubeecg, Cardioline S.p.A., Milan, Italy) monitored heart rate and detected any arrhythmias, while blood pressure was measured manually at rest, every 2 min during exercise, and during recovery [63].

### 2.4. Assessment of Physical Activity, Smoking, and Stress Perception

- Physical activity (weekly physical activity volume) was assessed by the short version of the International Physical Activity Questionnaire [48,57,59,63,64], which focuses on intensity (nominally estimated in metabolic equivalents—METs—according to the type of activity) and duration (in minutes) of physical activity. We considered the following levels: brisk walking (≈3.3 METs), activities of moderate intensity (≈4.0 METs) and activities of vigorous intensity (≈8.0 METs). In accordance with current guidelines [49], these levels were used to assess exercise volume, using the following equations:
  - Brisk walking (MET·min/week) = 3.3 × min of brisk walking × days of brisk walking;
  - Moderate intensity (MET·min/week) = 4.0 × min of moderate intensity activity × days of moderate intensity activities;
  - Vigorous intensity: (MET·min/week) = 8.0 × min of vigorous intensity activity × days of vigorous intensity activity;
  - METsTOT (Total weekly physical activity volume) (MET·min/week) = sum of brisk walking + moderate + vigorous MET·min/week scores;
  - METsMV (Total weekly physical activity volume of moderate and vigorous exercise) (MET·min/week) = sum of moderate + vigorous MET·min/week scores.

- Perception of somatic symptoms (short 4SQ), fatigue, and stress were guessed using a self-administered questionnaire [30,31,32,65,66] providing ordinal self-rated Likert scales from 0 (“very good”) to 10 (“very bad”) for each measure. The short 4SQ considers four somatic symptoms; thus, the total score, equal to the sum of the 0–10 scores on the single somatic symptom scales, ranged from 0 to 40.
- Smoke behavior: all subjects who reported having never smoked or had stopped smoking for more than one year were considered non-smokers.

### 2.5. Statistical Analysis

Data are presented as mean ± standard deviation (SD), indicating 95% confidence. Differences between the two considered groups were assessed with the Independent-Samples Mann–Whitney U Test and with GLM univariate considering sex and age as covariates, indicating effect size (Cohen’s d) as well. The relatively small number of subjects required consideration, keeping in mind the feasibility of the study. Although it is not a barrier in itself, it calls, however, for careful interpretation, avoiding generalizations and preferring methods (such as Cohen’s d) that are relatively unaffected by the number of subjects and focusing on the magnitude (small = 0.2; medium = 0.5; large = 0.8; very large = 1.3), not on the probability of an effect [67]. Spearman’s rank correlation coefficients were also computed, and their significance was tested. The Chi-Square test was also employed. We also employed Linear Regression analysis, considering VO_2max_ as the independent variable. Computations were performed with a commercial package (IBM SPSS 29), considering *p* < 0.05 as the significance threshold.

## 3. Results

Table 1 reports the descriptive data for the entire group of subjects and for the female and male subgroups. As expected, we noted significant differences regarding anthropometric, metabolic, and CPX data, as well as fatigue and symptom perceptions, in females as compared to males, underlying the importance of sex.

Table 2 reports a simple correlation matrix of age, parameters derived from the cardiopulmonary test, volume of physical activity, stress perception scores, and metabolic parameters. As expected, age negatively correlates with VO_2max_, and positively correlates with anthropometric and metabolic parameters. Despite the relatively small population, VO_2max_ negatively correlates with stress perception (*p* = 0.03), perception of fatigue (*p* < 0.001) and somatic symptoms (*p* < 0.001); as expected, it also correlates with fat mass percentage (*p* < 0.001), fasting plasma glucose level, total and LDL cholesterol; it correlates positively with METsTOT (total weekly physical activity volume) (*p* < 0.001) and METsMV (total weekly physical activity volume of moderate and vigorous exercise) (*p* < 0.001). These two measures of physical activity volume also correlate well with perception of stress, fatigue, and somatic symptoms. Perception of fatigue and of somatic symptoms directly correlate with FM% (respectively, *p* = 0.005 and *p* = 0.003).

Table 3 reports linear regression analysis, considering VO_2max_ as the independent variable, which shows a significant result for all the considered variables with the exception of HDL cholesterol and triglycerides.

Table 4 reports anthropometric, hemodynamic, and metabolic data, parameters derived from cardiopulmonary test and stress perception scores. Significance derived from the general linear model (GLM) univariate considering sex and age as covariates was reported (considering the importance of these two factors, as highlighted by the result reported in Table 1 and Table 2), as well as significance derived from the Independent-Samples Mann–Whitney U Test.

**Figure 1:**
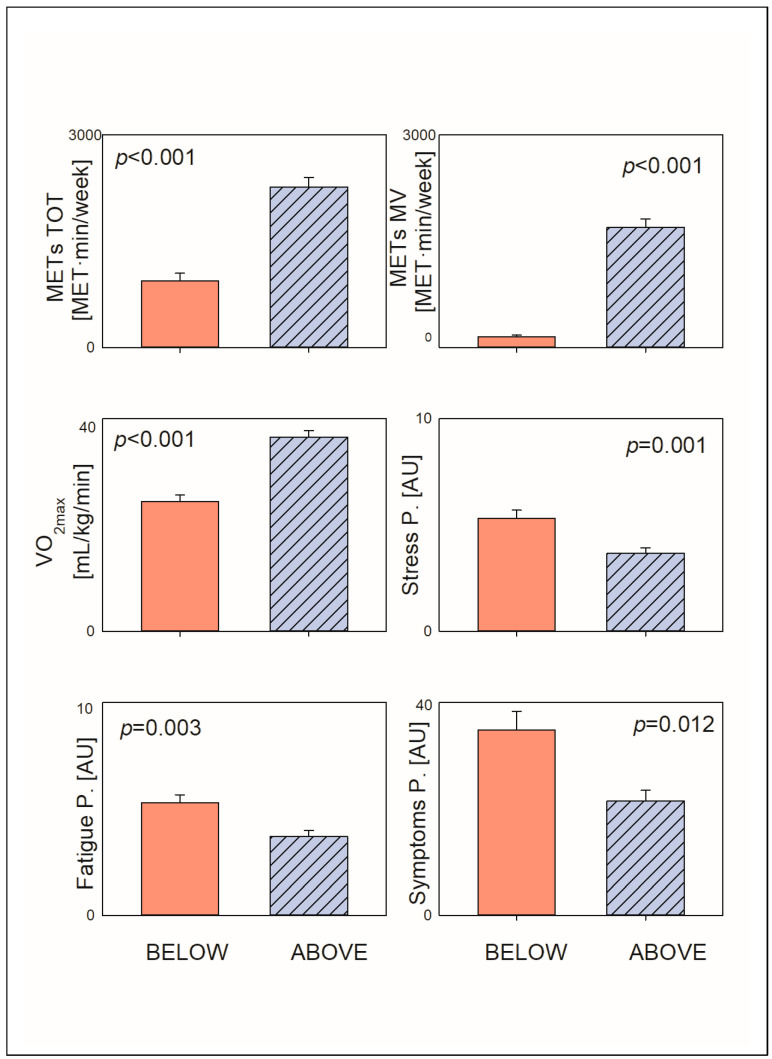
Differences in the main selected parameters between subjects who spontaneously met (ABOVE) current physical activity goals and those who did not (BELOW), considering the total weekly volume of moderate and vigorous physical activity. Physically active subjects showed better cardiorespiratory fitness (V0_2max_), reduced perception of stress, fatigue, and somatic symptoms.

Age was significantly different in the two considered groups, respectively, 40.97 ± 12.07 in group BELOW and 32.34 ± 12.50 in group ABOVE (95 C.I. 0.35, 1.04 *p* < 0.001). The percentage of smoking subjects was similar in the two considered groups, respectively, n = 10 (17.24%) in group BELOW, and n = 10 (11.49%) in group ABOVE (Chi Square test: ns); likewise, no significant differences were noted regarding lipid profile, fasting plasma glucose, arterial pressure values, and heart rate in basal condition (see Table 1). As expected, BMI and FM% were greater in the group BELOW, while VO_2max_, HR/VO_2_, and HR at threshold were significantly greater in the group ABOVE as indicated by both parametric and non-parametric statistics (Figure 1). Interestingly, both the employed statistical methodologies (Independent-Samples Mann–Whitney U Test and with GLM univariate considering sex and age as covariates) reveal that the perception of stress, fatigue, and somatic symptoms was significantly greater in the BELOW group (see Table 1).

## 4. Discussion

In this paper, we observed a significant correlation between VO_2max_ (the direct measure of CRF assessed through cardiopulmonary stress testing) and perceptions of stress, fatigue, and somatic symptoms. Additionally, we found that inactive individuals who do not meet the physical activity goals outlined by WHO guidelines [48], are characterized by higher perceptions of stress, fatigue, and symptoms compared to those who are physically active.

The connection between stress and exercise is gaining increasing importance in clinical practice, especially considering a bidirectional relationship between stressful conditions and physical activity [22,56]. The presence of psychosocial stressors may limit reaching high sport performance in elite athletes [56,68], while in patients or in non-athletes, who need to perform exercise to improve health, it may represent a real barrier, which reduces the motivation to exercise [69]. On the other hand, many pieces of scientific evidence show that exercise represents a fundamental tool to manage chronic stress [39,40,42,70] and foster well-being, and nowadays it is one of the main behavioral strategies employed in this regard, even in patients characterized by psychiatric conditions such as depression and serious anxiety [71]. Exercise may counterbalance many alterations in control mechanisms due to chronic stress, such as inflammation, increased release of some hormones, and autonomic nervous control derangement (particularly sympathetic overactivity [72,73] and improve neuromodulation of the brain [74,75]. Moreover, stressful conditions may facilitate functional syndromes, such as chronic stress syndrome, fibromyalgia, and chronic fatigue syndrome, all characterized by the presence of somatic symptoms not explained by conventional diseases [76,77] and by impairments in control mechanisms.

Aerobic exercise and stressful conditions act on the same control mechanisms [76], but with opposite results. While chronic stress induces changes in control mechanisms, which, in the long run, may determine an increased risk of developing chronic diseases (close to genetic and behavioral aspects) and a reduction in well-being, aerobic exercise may reduce inflammation, enhance autonomic control, and optimize hormonal stress response [75], leading to a lower risk of developing chronic conditions, improving their prognosis [75], or promoting well-being. Of particular interest may be the role of functional connectivity among several intrinsic brain networks [78]; in adults, maladaptive stress responses are linked to functional and structural changes in brain networks [79], and higher perceived stress is associated with disrupted communication within brain networks [80]. Even stress-induced cortisol levels are connected with changes in brain connectivity [81]. Among lifestyle behaviors capable of positively affecting brain connectivity, exercise plays a pivotal role [82]; CRF is, in fact, related to specific brain networks considered relevant to age-related cognitive changes and the risk of developing psychiatric and neurological diseases [83]. The scientific literature suggests that unhealthy behaviors, particularly sedentariness, may accelerate brain aging, while exercise and high CRF may counterbalance cognitive decline, supporting the hypothesis that aerobic exercise has apparent neuroprotective effects [77] through mechanisms such as improved cerebral blood flow, reduced inflammation, and enhanced neuroplasticity [77], which represent the brain’s ability to structurally and functionally adapt to changing demands, including remodeling of existing synapses to optimize neural communication [84]. Other important mechanisms linking exercise to improved brain function need to be mentioned; one of these is the role of Exerkines (signaling molecules such as hormones, metabolites, proteins, lipids, and nucleic acids) released by peripheral tissues in response to exercise, which exert their effects on the brain through endocrine, paracrine, or autocrine pathways [77]. Another factor is the action of gut microbiota, which produces a multitude of metabolites crucial for maintaining brain health; it is improved by regular moderate exercise [85], and its composition and diversity correlate with CRF.

It is of paramount importance that the exercise modality and volume employed are the ones capable of positively affecting those control mechanisms and brain circuits.

Aerobic exercise performed at the tailored volume (considering intensity, duration, and frequency) meets those characteristics, while anaerobic exercise (endurance exercise performed at a very high intensity or strength exercise not performed at low intensity) can have counterproductive effects, worsening inflammation, inducing the release of excess stress hormones, and provoking autonomic imbalance, in particular parasympathetic overactivity [43,86]. Aerobic training is the most important determinant of the increase in CRF both in patients and in healthy subjects [3,4,5,7,11,12,86]. Notably, multicomponent exercise, such as tai chi or yoga, which adds the modulation of breathing [48] to the aerobic/flexibility/strength modality of exercise (usually performed at low–moderate intensity), has an essential relaxing effect due to the improvement in parasympathetic activity [49,50] and other neuropsychological functions [51] mediated by elicited reflex started by controlled breathing. These mechanisms also characterize mindfulness, a technique that nowadays represents a cornerstone of sport psychology, particularly useful in the management of stress in athletes [51].

Cardiorespiratory fitness represents a strong and consistent predictor of mortality among adults [2]. The possibility of measuring CRF by direct assessment represents, ideally, the best manner to assess the efficacy of aerobic exercise training, when the specific goal of the training is to improve bodily control systems, those associated with cardiometabolic risk and impaired by chronic stress. In this paper, we showed a correlation between CRF (directly assessed by VO_2max_) and the perception of stress, fatigue, and somatic symptoms, corroborating the observation of the important link between stress and aerobic exercise. Notably, it may reinforce the data that CRF and mortality risk are influenced by genetic and behavioral/social confounders that may differ between active and non-active people [6].

In this paper, we also employed the most indirect assessment of CRF, as indicated by the quantification of exercise volume using a validated questionnaire [57]. We observed not only an expected correlation between exercise volume and VO_2max_ but also a correlation between exercise volume and perception of stress, fatigue, and somatic symptoms, underlining, again, the important link between stress perception and exercise capacity.

In the present study, we assessed the perception of stress and fatigue and other somatic symptoms, using simple scales that we had already employed in other studies of our group [30,31,32,49,50,65,66,69]. The assessment of stress perception is widely employed in research settings to study the construct of stress. Many definitions of stress exist, and in general, it may be considered the psychological, behavioral, and physical consequences of the relationship between a subject and a source of stress, and it occurs when demands exceed the individual’s capability to cope [76]. Some research has focused on the assessment of the causes of stress, situations that can evoke emotional, cognitive, or biological reactions leading to a stress response. Considering that these situations may differ significantly across individuals [87], we preferred to focus on individual perceptions of stress and stress-related somatic symptoms, focusing on people’s subjective experience rather than objective stressors. We assessed the perception of stress, fatigue, and stress-related somatic symptoms using a self-administered questionnaire, providing nominal self-rated scales (higher values indicate higher degrees of perception) from 0 (‘no perception’) to 10 (‘highest perception’) for each measure. This methodology might be simplistic; nevertheless, scores correlate with physiological parameters such as markers of autonomic controls [32,49], and it is very simple to apply. In previous papers, we observed that in young adults, stress perception was higher than in older ones [88], and it was also reduced in athletes (characterized by both low–medium static and dynamic components) who were eventually selected for the Rio 2016 Olympic Games Italian team as compared to those who were not [89]; stress perception was also reduced in elite rowing athletes who exhibited high performance (gold medal at word championship) [90]. We also observed that stress perception was lower after the aerobic training program [91]. In this paper, this simple method was able to reveal a significant correlation with both CRF and weekly exercise volume. Moreover, it also unveiled a significant correlation between the perception of fatigue and somatic symptoms with FM% (respectively, *p* = 0.005 and *p* = 0.003); interestingly, this association was evident only considering the perception of symptoms and not the perception of stress, which refers more to a cognitive understanding about stress itself rather than its consequences on bodily perception, corroborating the recent data present in the literature [92] on a large population which shows a significant link between weight over-perception and perceived stress, highlighting the important role of stressful conditions in overweight issues [35,92,93].

It is also important to comment on the role of stressful conditions in impacting physical activity, nutrition, and sleep habits [69]. They may represent an essential barrier to exercising, reducing motivation to adopt healthy behavior and fostering unhealthy ones; they frequently promote smoking and food-seeking behavior (but may also cause a loss of appetite), inducing individuals to choose progressively more palatable food [94,95]; they promote sedentary life, the feeling of “lack of time” to exercise, and the desire to rest (but also, in selected individuals, to perform high volume/intensities of exercise). Some preclinical and clinical studies [94,95,96] are disentangling a critical mechanism responsible for these behavioral choices: the seeking of pleasure or “gratification”, choosing behaviors that interfere with the brain reward systems.

Briefly, exercise may be considered a convenient tool to buffer the stress effects of mechanisms that determine health and disease [46]; indeed, exercise has been shown to favor positive changes in strategy to cope with stressful events [46,97] and to foster socialization, enjoying time [98] and quality of life [46], suggesting that exercise benefits may be linked to both physiological and psychosocial mechanisms [99] essential to manage stress not only in selected patients but also in healthy people who have to cope with daily stress [46].

Limitations: We must acknowledge some limitations of our study. First, the study population consisted of only 145 subjects, which does not permit any generalization of the results. Although this is not a major barrier by itself, it requires careful interpretation and the avoiding of broad generalizations. To address this issue, we chose to use statistical methods (such as Cohen’s d) that are less affected by the number of subjects, focusing on the magnitude (small = 0.2; medium = 0.5; large = 0.8; very large = 1.3), rather than the probability of an effect.

Second, this study is a simple observational, retrospective study. It is not an international clinical trial, and it was not designed at the moment of the subjects’ assessment with a specific research goal.

Third, we did not assess mechanisms underlying the link between CRF and stress, such as immunological, endocrine, and autonomic functions; nevertheless, the scientific literature has already thoroughly illustrated their importance in mediating exercise and stress effects. In particular, it is crucial to focus on the role of stress in influencing the autonomic nervous system and the role of aerobic exercise in enhancing autonomic nervous system control.

Fourth, we did not directly assess weekly exercise volume using wearable devices; however, the questionnaire used was validated [57] and widely adopted, showing good reliability and validity values (Typical IPAQ correlations were about 0.80 for reliability and 0.30 for validity) [57].

Fifth, we assessed only the perception of stress and symptoms possibly related to stressful conditions; we did not perform an exhaustive assessment of mental outcomes, which were indeed outside the goal of the present study.

Practical implications: This study may help the understanding of the complex relationship between cardiorespiratory fitness and stress perception. This link may both reinforce the choice of exercise as a tool to counterbalance stress in some individuals/patients who report perceiving the adverse effects of it, and the choice of other stress management strategies (such as mindfulness, cognitive behavioral therapy, etc.) in subjects (particularly athletes) who experience a reduction in physical performances due to stress. It may also contribute to the role of behavioral/social confounders (such as stress) as determinants of cardiorespiratory fitness.

Moreover, this study evidences a clear correlation between VO_2max_ (the ideal method to assess cardiorespiratory fitness) and the volume of exercise calculated by the questionnaire, an indirect but economically and organizationally more sustainable tool, to estimate physical training in a clinical setting. The insights gained from this study may be valuable for generating hypotheses that can be further explored in future studies with larger, more representative samples.

## 5. Conclusions

In conclusion, this research observed a link between cardiorespiratory fitness, directly measured by VO_2max_, and perceptions of stress, fatigue, and somatic symptoms in a healthy population, highlighting the important connection between stress and exercise. This evidence might, on the one hand, corroborate the role of exercise as a tool to manage stress and, on the other hand, focus on the role of stress as a possible determinant of CRF. This connection was further supported by the observation that subjects who did not meet the physical activity goals as indicated by WHO guidelines [48] reported higher perception of stress, fatigue, and symptoms compared to physically active subjects. These data may contribute to the scientific evidence supporting the use of aerobic exercise as a behavioral strategy to manage stress, improve well-being, prevent chronic non-communicable diseases, and promote healthy aging. These data might be a valuable “starting point” for designing future investigations involving a large study population, including assessments of immunological, autonomic, and metabolic controls. Such investigations are essential to confirm the results presented in this study.

## Figures and Tables

**Table 1:** Individual anthropometrics, hemodynamics, CPX, and stress perception parameters in all subjects, female and male. Data are expressed as median ± SD; *p* = 0.05 females vs. males. Abbreviations: FM% = fat mass percentage; FFM% = fat free mass percentage; TOT Chol = total cholesterol; HDL = high-density lipoprotein; LDL = low-density lipoprotein; FPG = fasting plasma glucose; SAP = systolic arterial pressure; DAP = diastolic arterial pressure; HR thres. = heart rate at threshold; HR max: maximum heart rate; VO_2max_ = maximal oxygen consumption; RQ = respiratory quotient; VO_2_/Work = oxygen consumption per work (expressed in watts); HR/VO_2_ = rate of change in heart rate (HR) relative to the rate of change in oxygen consumption (VO_2_); VE/VCO_2_ = ratio of minute ventilation (VE) to carbon dioxide production (VCO_2_); Mets = metabolic equivalents of task; M-V = moderate-vigorous; P. = perception; AUs = arbitrary units. 4SQ = short somatic symptoms stress-related questionnaire. Significant values are written in bold.

Variable		All	Females	Males	Mann–Whitney U
		Mean ± S.D	Mean ± S.D	Mean ± S.D	Sign. *p* =
Age	[yrs]	35.79 ± 13.00	38.04 ± 12.30	32.21 ± 25.00	**0.006**
BMI	[kg/m^2^]	25.72 ± 5.50	26.14 ± 6.06	25.05 ± 4.46	0.609
Waist	[cm]	87.96 ± 14.30	87.86 ± 15.71	88.13 ± 11.87	0.542
FM%	%	27.45 ± 11.17	33.27 ± 8.70	18.04 ± 7.82	**0.000**
FFM%	%	72.55 ± 11.17	66.73 ± 8.70	81.96 ± 7.82	**0.000**
TOT Chol.	[mg/dL]	210.36 ± 40.65	214.42 ± 40.04	194.94 ± 41.01	0.159
HDL Chol	[mg/dL]	58.89 ± 14.32	61.43 ± 13.27	50.60 ± 14.94	**0.004**
LDL Chol	[mg/dL]	130.16 ± 38.48	133.49 ± 36.00	119.27 ± 45.30	0.231
Triglycerides	[mg/dL]	100.76 ± 47.45	96.27 ± 36.76	115.38 ± 71.88	0.497
FPG	[mg/dL]	88.67 ± 9.92	87.88 ± 10.29	91.13 ± 8.52	0.106
SAP	[mmHg]	115.30 ± 10.76	113.53 ± 12.25	118.11 ± 7.07	**0.000**
DAP	[mmHg]	70.34 ± 9.44	70.61 ± 9.97	69.91 ± 8.61	0.817
HR thres.	[beat/min]	125.25 ± 19.22	122.95 ± 18.36	128.84 ± 20.14	0.143
HR max	[beat/min]	163.77 ± 16.61	160.77 ± 16.57	168.51 ± 15.67	**0.007**
VO_2max_	[mL/kg/min]	31.65 ± 12.30	25.75 ± 9.16	40.88 ± 10.87	**0.000**
RQ	.	1.16 ± 0.10	1.16 ± 0.10	1.15 ± 0.09	0.682
VO_2_/Work	[mL/min/Watt]	9.81 ± 3.21	8.26 ± 2.61	12.16 ± 2.54	**0.000**
HR/VO_2_	[bpm/L/min]	2.96 ± 0.84	3.46 ± 0.87	2.54 ± 0.55	**0.000**
VE/VCO_2_	.	23.85 ± 3.65	24.45 ± 3.93	22.92 ± 2.99	**0.014**
Mets M-V	.	1080.69 ± 1139.51	856.18 ± 1080.16	1437.50 ± 1149.65	**0.000**
TOT. Mets	.	1737.39 ± 1266.54	1461.12 ± 1184.00	2176.46 ± 1279.50	**0.000**
Stress P. score	[AU]	4.33 ± 2.79	4.69 ± 2.84	3.77 ± 2.64	0.062
Fatigue P. score	[AU]	4.33 ± 2.83	4.80 ± 2.76	3.59 ± 2.80	**0.011**
4SQ score	[AU]	26.79 ± 23.44	31.85 ± 22.56	18.73 ± 22.73	**0.000**

**Table 2:** Spearman’s correlation matrix of age, parameters derived from cardiopulmonary test, volume of physical activity, stress perception scores, and metabolic parameters. ** Correlation is significant at the 0.01 level. * Correlation is significant at the 0.05 level. Abbreviations: VO_2max_ = maximal oxygen consumption; METs = metabolic equivalents of task; M-V = moderate to vigorous; P. = perception; 4SQ = short somatic symptoms stress-related questionnaire; Chol.= cholesterol; Trig. = triglycerides, FPG = fasting plasma glucose. Significant values are written in bold.

	AGE	VO2max	METs Total	METs M-V	Stress P. Score	Fatigue P. Score	4SQ Score	Fat Mass%	Total Chol.	HDL Chol.	Triglycerides	FPG
AGE	1											
VO_2max_	−0.552 **	1										
METs Total	−0.150	0.461 **	1									
METs M-V	−0.235 **	0.488 **	0.848 **	1								
Stress P. Score	0.014	−0.183 *	−0.275 **	−0.243 **	1							
Fatigue P. Score	0.036	−0.321 **	−0.275 **	−0.235 **	0.720 **	1						
4SQ Score	0.136	−0.302 **	−0.255 **	−0.216 **	0.555 **	0.623 **	1					
Fat Mass%	0.599 **	−0.823 **	−0.358 **	−0.346 **	0.168	0.241 **	0.253 **	1				
TOT Chol.	0.316 **	−0.351 **	0.007	−0.124	0.105	0.051	0.120	**0.239** *	1			
HDL Chol.	−0.343 **	0.142	0.000	−0.096	0.103	0.099	0.116	−0.182	0.025	1		
Triglycerides	0.299 *	−0.202	−0.149	−0.172	−0.123	−0.077	−0.060	0.087	0.418 **	−0.349 **	1	
FPG	0.286 *	−0.246 *	−0.228	−0.177	0.208	0.331 **	0.194	0.275 *	0.019	−0.226	0.015	1

**Table 3:** Linear regression analysis considering VO_2max_ as the independent variable and age, physical activity, stress perception scores and metabolic parameters as dependent variables. Abbreviations: VO_2max_ = maximal oxygen consumption; METs = metabolic equivalents of task; M-V = moderate to vigorous; P. = perception; 4SQ = short somatic symptoms stress-related questionnaire; Chol. = cholesterol; Trig. = triglycerides, FPG = fasting plasma glucose. Significant values are written in bold.

	AGE	METs Total	METs M-V	Stress P. Score	Fatigue P. Score	4SQ Score	Fat Mass%	Total Chol.	HDL Chol.	Triglycerides	FPG
Regression coefficient	−0.584	47.95	45.77	−0.042	−0.074	−0.581	−0.742	−1.25	0.179	−0.837	−0.211
Significance	<**0.001**	<**0.001**	<**0.001**	**0.030**	<**0.001**	<**0.001**	<**0.001**	**0.003**	0.264	0.099	<**0.001**

**Table 4:** Individual anthropometrics, hemodynamics, CPX, and stress perception parameters in subjects who do not reach the WHO physical activity goals (Below Group) and subjects who reach them (Above Group). Data are expressed as median ± SD; *p* = 0.05 Below vs. Above; Cohen’s d uses the sample standard deviation of the mean difference. It is not affected by the number of subjects and rather focuses on the magnitude of an effect. A value > 0.5 suggests a meaningfully medium effect, while a value > 0.8 suggests a meaningfully large effect [55]. Abbreviations: C.I. = confidence interval; FM% = fat mass percentage; FFM% = fat free mass percentage; TOT Chol = total cholesterol; HDL = high-density lipoprotein; LDL = low-density lipoprotein; FPG = fasting plasma glucose; SAP = systolic arterial pressure; DAP = diastolic arterial pressure; HR thres. = heart rate at threshold; HR max: maximum heart rate; VO_2max_ = maximal oxygen consumption; RQ = respiratory quotient; VO_2_/Work = oxygen consumption per work (expressed in watts); HR/VO_2_ = rate of change in heart rate (HR) relative to the rate of change in oxygen consumption (VO_2_); VE/VCO_2_ = ratio of minute ventilation (VE) to carbon dioxide production (VCO_2_); Mets = metabolic equivalents of task; M-V = moderate–vigorous; P. = perception; AUs = arbitrary units. 4SQ = short somatic symptoms stress-related questionnaire. Significant values are written in bold.

Variable		BELOW Group	ABOVE Group	Cohen’s d	Univariate GLM (Covariates: Sex, Age)		Mann–Whitney U
		Mean ± S.D	Mean ± S.D		(95% C.I.)	Sign. *p* =	Sign. *p* =
BMI	[kg/m^2^]	27.75 ± 6.90	24.37 ± 3.81	**0.644**	**(0.31, 3.86)**	**0.022**	**0.005**
Waist	[cm]	91.55 ± 17.37	85.43 ± 11.09	0.437	(−0.59, 9.23)	0.084	0.072
FM%	%	32.27 ± 10.93	23.86 ± 10.00	**0.809**	(−0.45, 4.48)	0.108	**0.000**
FFM%	%	67.72 ± 10.92	76.14 ± 10.00	**0.809**	(−4.48, 0.45)	0.108	**0.000**
TOT Chol.	[mg/dL]	213.90 ± 41.06	205.48 ± 40.27	0.207	(−16.60, 22.14)	0.776	0.531
HDL Chol	[mg/dL]	60.14 ± 14.64	57.23 ± 1 3.99	0.199	(−3.67, 9.40)	0.384	0.507
LDL Chol	[mg/dL]	132.52 ± 37.26	127.12 ± 40.47	0.140	(−16.91, 19.33)	0.894	0.882
Triglycerides	[mg/dL]	102.00 ± 36.47	99.10 ± 59.82	0.061	(−21.28, 24.07)	0.902	0.236
FPG	[mg/dL]	90.23 ± 10.23	86.46 ± 9.21	0.390	(−0.89, 8.61)	0.109	0.078
SAP	[mmHg]	114.55 ± 12.12	115.80 ± 9.8	0.115	(−5.79, 1.52)	0.250	0.621
DAP	[mmHg]	72.15 ± 10.23	69.13 ± 8.72	0.324	(68.08, 72.31)	0.863	**0.031**
HR thres.	[beat/min]	115.42 ± 16.17	131.92 ± 18.31	**0.944**	(−**17.49,** −**5.73**)	**0.000**	**0.000**
HR max	[beat/min]	156.89 ± 16.88	168.38 ± 16.61	**0.732**	(−9.32, 0.13)	0.056	**0.000**
VO_2max_	[mL/kg/min]	24.46 ± 9.30	36.53 ± 11.72	−**1.117**	(−**8.84,** −**3.04**)	**0.000**	**0.000**
RQ	.	1.17 ± 0.09	1.15 ± 0.10	0.211	(−0.01, 0.06)	0.124	0.192
VO_2_/Work	[mL/min/Watt]	8.39 ± 2.74	10.77 ± 3.21	**0.792**	(−1.78, 0.09)	0.076	**0.000**
HR/VO_2_	[bpm/L/min]	3.5 ± 1.08	2.76 ± 0.65	**0.939**	**(0.24, 0.99)**	**0.002**	**0.006**
VE/VCO_2_	.	24.75 ± 4.21	23.25 ± 3.11	0.418	(−0.35, 2.42)	0.143	**0.042**
Mets M-V	.	152.07 ± 210.66	1699.77 ± 1084.21	**1.818**	(−**1805.80,** −**1186.00**)	**0.000**	**0.000**
TOT. Mets	.	939.80 ± 874.02	2269.11 ± 1210.21	**1.221**	**(1647.26, 862.51)**	**0.000**	**0.000**
Stress P. score	[AU]	5.33 ± 2.93	3.67 ± 2.50	**0.620**	**(0.73, 2.67)**	**0.001**	**0.001**
Fatigue P. score	[AU]	5.28 ± 2.87	3.70 ± 2.63	**0.577**	**(0.50, 2.47)**	**0.003**	**0.001**
4SQ score	[AU]	34.81 ± 26.98	21.44 ± 19.10	**0.592**	**(2.29, 18.42)**	**0.012**	**0.003**

## Data Availability

The dataset used in this study will be uploaded to the Zenodo repository. Requests to access the dataset should be directed to daniela.lucini@unimi.it.

## Abbreviations

The following abbreviations are used in this manuscript:

AU	Arbitrary Units
BIA	Bioelectrical Impedance Analysis
BMI	Body Mass Index
CI	Confidence Interval
CNCD	Chronic Non-Communicable Diseases
CRF	Cardio Respiratory Fitness
CPX	Cardiopulmonary exercise test
DAP	Diastolic Arterial Pressure
ECG	Electrocardiogram
FM	Fat Mass
FFM	Free Fat Mass
GLM	General Linear Model
HDL	High Density Lipoprotein
HR	Heart Rate
IPAQ	International Physical Activity Questionnaire
IRCCS	Istituto di Ricovero e Cura a Carattere Scientifico (Scientific Institute for Research, Hospitalization and Healthcare)
LDL	Low Density Lipoprotein
METs	Metabolic Equivalents
METsMV	Moderate and Vigorous Physical Activity Volume Expressed in METs
SAP	Systolic Arterial Pressure
SD	Standard Deviation
4SQ	Short Somatic symptoms Stress-related Questionnaire
RQ	Respiratory Quotient
VE/VCO_2_	Ratio of Minute Ventilation (VE) to Carbon Dioxide Production (VCO2)
VO_2_	Oxygen Consumption
VO_2max_	Maximum Rate of Oxygen Consumption per Kilogram of Body Mass
